# Chemogenetic Silencing of Neonatal Spontaneous Activity of Projection Neurons in the Dorsal Striatum of Mice

**DOI:** 10.21769/BioProtoc.5088

**Published:** 2024-10-20

**Authors:** Bojana Kokinovic, Maria Ryazantseva, Svetlana Molchanova

**Affiliations:** 1 Molecular and Integrative Biosciences Research Programme, Faculty of Biological and Environmental Sciences, University of Helsinki, Helsinki, Finland; 2 Department of Pharmacology, Faculty of Medicine, University of Helsinki, Helsinki, Finland

**Keywords:** Neonatal virus transduction, hM4Di, CNO, Activity suppression, Synaptic development

## Abstract

Neuroscience incorporates manipulating neuronal circuitry to enhance the understanding of intricate brain functions. An effective strategy to attain this objective entails utilizing viral vectors to induce varied gene expression by delivering transgenes into brain cells. Here, we combine the use of transgenic mice, neonatal transduction with adeno-associated viral constructs harboring inhibitory designer receptor exclusively activated by designer drug (DREADD) gene, and the DREADD agonist clozapine N-oxide (CNO). In this way, a chemogenetic approach is employed to suppress neuronal activity in the region of interest during a critical developmental window, with subsequent investigation into its effects on the neuronal circuitry in adulthood.

Key features

• Comprehensive protocol for newborn viral transduction in the dorsal striatum of mice

• Uses a viral construct encoding inhibitory DREADD under the control of Cre recombinase to attenuate the activity of specific cell types in the brain

## Graphical overview


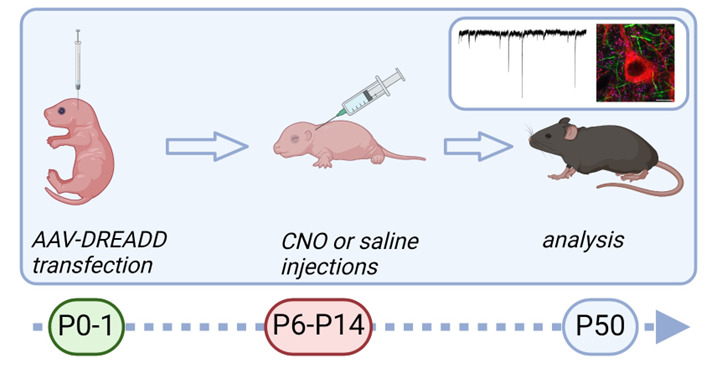


**Graphic overview of the procedure for chemogenetic silencing of striatal neurons in developing mouse brain.** Newborn pups (first or second day of life) are injected with adeno-associated virus (AAV) coding inhibitory designer receptors exclusively activated by designer drugs (DREADD) to the dorsal striatum. Starting from postnatal day 6, pups are injected with clozapine N-oxide (CNO) subcutaneously twice a day for 9 consecutive days. Analysis of the consequences can be performed at the adult age.

## Background

Using neonatal animals in scientific research allows the investigation of developmental processes. This is especially important for studying the maturation of neuronal networks. Time-limited critical periods of brain development are of particular interest due to their possible role in the origin of neuropsychiatric disorders [1]. Therefore, the approaches of time-specific alteration of neuronal activity within different brain areas are needed for this kind of research. Chemogenetic manipulations are widely used for time-limited modulation of neuronal activity in vivo. Here, we designed the protocol to suppress the activity of striatal projection neurons during the second week of life (P6–P14) to study the activity-dependent development of long-range GABAergic projections. To achieve this aim, we performed viral injections to the striatum of newborn (P0–2) mice with minimal surgery and under inhalation anesthesia, which ensures a high survival rate for the pups.

The current protocol for viral injections followed by designer receptors exclusively activated by designer drugs (DREADD)-agonist administration (CNO, clozapine-N-oxide dihydrochloride) was adapted from two previously published protocols [2] and [3]. As demonstrated by [2], inhalation-based anesthesia with isoflurane for early postnatal (P0−2) mice significantly enhances animal welfare. In our protocol, the skin and skull were gently perforated with a needle, which was later used to deliver the viral constructs. Alternatively, the skin and skull can be carefully perforated by a G30 syringe needle, and a glass pipette attached to a Hamilton syringe can be used to deliver the virus instead of a needle. This alternative is preferable when targeting smaller brain areas, such as the amygdala [4]. This needle-based or pipette-based injection through the skin and skull enhanced the survival of operated pups, because of the absence of sutures, which usually triggers infanticide. Although our protocol was designed for injections into the dorsal striatum, it can be easily adapted to another brain area of neonatal mice.

The following stereotaxic coordinates for viral injections in the striatum were determined in reference to the vascular lambda: AP 2.4; ML ±1.2; DV 2.3 and 2.7. The injections were done bilaterally and at two coordinates in the DV axis. The needle was not removed between the first and the second DV coordinate. The amount of the virus injected into each coordinate was 150 nL (left: 150 nL + 150 nL; right: 150 nL + 150 nL). Coordinates were based on those previously published [3]. If the coordinates for the brain area of interest are not known, the “Developing Mouse Brain Atlas” can be used to suggest the coordinates for injections in neonates. The suggested coordinates can then be checked by injecting a tissue dye under terminal anesthesia, followed by brain removal and slicing with the vibratome to verify coordinates with the dye signal. The brain can also be fixed with 4% PFA before checking the coordinates.

The injections of CNO were performed subcutaneously twice a day (9:00 and 18:00). Starting from P6, mice were injected with CNO dissolved in sterile saline solution (0.9% NaCl) or saline alone consecutively for 9 days. The administration method was chosen to prolong the effective time of the drug without sharply reaching the maximal concentration of the drug in the brain. It takes longer for the concentration of small molecules to reach maximal levels in the interstitial brain fluid when they are injected subcutaneously compared to intraperitoneally injected [5,6]. We used the CNO treatment of wild-type animals not expressing hM4Di as a control to verify that CNO itself did not cause any detectable effects. Alternatively, other muscarinic-based DREADD agonists can be used, such as Compound 21, deschloroclozapine, JHU37152, and JHU37160 [7–10]. These more modern alternative agonists differ from CNO in their pharmacological properties and generally demonstrate less off-target effects [7–9]. In this case, an appropriate dosage and an administration protocol should be defined separately. It is advisable to check if the compound was previously used in research related to the experimental objectives. This can aid in the design and refinement of the protocol and help in mitigating off-target effects if any have been previously documented. To assess potential off-target effects, administration of the compound to wild-type animals lacking DREADD expression can be used.

## Materials and reagents

**Biological materials**

1. Mice: Oprm1-2A-Cre (Oprm1-Cre [11]),
2. Virus for DREADD: pAAV-hSyn-DIO-HA-hM4D(Gi)-IRES-mCitrine (a gift from Bryan Roth, Addgene viral prep # 50455-AAV8, http://n2t.net/addgene:50455; RRID: Addgene_50455)

**Reagents**

1. Clozapine-N-oxide dihydrochloride (HelloBio, catalog number: HB6149)
2. Sterile 0.9% NaCl (Fresenius Kabi AG, catalog number: 936808)
3. Isoflurane (1000 mg/g) (Attane vet 1000 mg/g) (Primal Critical Care, catalog number: 170579)
4. 70% EtOH (Anora Industrial, ETAX, catalog number: 1025904)
5. Hydrogen peroxide 1% (Pharmacare)
6. Glycerol 99% (Sigma-Aldrich, catalog number: G6279) or sterile PBS (Sigma-Aldrich, catalog number: P2272)
7. Green dye: Fast green FCF (Sigma-Aldrich, catalog number: F-7262)

**Solutions**

1. CNO solution (see Recipes)
2. Green dye in glycerol solution or PBS (see Recipes)

**Recipes**

1. **CNO solution**
  In a sterile 1.5 mL Eppendorf tube, measure the precise quantity of CNO powder and add sterile saline solution. Vortex well until the powder is totally dissolved. Aliquot the solution and store it at -20 °C.
  **Table:** 

Components	Final concentration	Quantity
CNO powder	30 mg/mL	15 mg
NaCl	9 g/L	500 μL
Total		~ 0.5 mL
2. **Green dye in glycerol solution or PBS**
  Use a sterile Eppendorf tube (0.2–1.5 mL), add 200 µL of glycerol (99%) or sterile PBS, add green dye powder for a final concentration of dye of 1%, and vortex well until it becomes green. Store the solution with PBS at 4 °C; the glycerol-based solution can be stored at room temperature.
  **Table:** 

Components	Final concentration	Quantity
Glycerol or PBS	99%	200 μL
Green dye powder	1%	2 mg
Total		200 µL

**Laboratory supplies**

1. NF33BV-2 33 ga. beveled NanoFil needle (World Precision Instruments, UK, catalog number: NF33BV-2)
2. NanoFil syringe 10 μL (World Precision Instruments, UK, catalog number: NANOFIL)
3. 200 μL pipette tip for making a mask (SARTORIUS, Safetyspace Tips, model: 790201)
4. T-style male luer fitting for tubing i.d. 1/8 in. to connect the mask and tubes (i.e., Sigma-Aldrich, catalog number: Z274240)
5. Scissors (World Precision Instruments, UK, catalog number: 500216)
6. Tape (Scotch Brand, Scotch® Magic™ 810, catalog number: 810-3PK-BXD)
7. Autoclavable bags (200 x 300 mm, Ratiolab, catalog number: 7001000)
8. Ice
9. Cotton buds
10. Eppendorf tube 1.5 mL (FisherBrand, catalog number: 05-408-129)
11. Eppendorf tube 0.2 mL (Nippon Genetics EUROPE GmbH, catalog number: FG-021)
12. Pipette 0.1–2 μL (Thermo Scientific™, F1-ClipTip™, catalog number: 4641310N)
13. Sterile filtered pipette tips 0.1–10 μL (SARTORIUS, Safetyspace filter tips, model: 790011F)
14. Hamilton syringe with male luer lock (25 μL), instrument syringe (Hamilton, model: 1702 TLLX, catalog number: 80222)
15. 30G syringe needles (hypodermic needle, Microlance^TM^ 3) (VWR, catalog number: 613-3942)
16. Blu Tack (Bostik, model: Blu Tack® Handy)
17. Wipes Kimberly-Clark^TM^ Kimtech Science^TM^ delicate task wipes (Fisher Scientific, Kimberly-Clark^TM^, catalog number: 7558)
18. Gloves
19. Tunnel for mice handling (https://3rc.org/refined-mouse-handling-overview/operations-and-tunnels), e.g., cardboard tube
20. PVC tubing 1/8 in. i.d. 1/4 in. o.d. (i.e., OC-TUBING, WPI, US)

## Equipment

1. Stereotaxic frame for mice (StoelTing, US, model: 51615M)
2. Temperature controller with heating pad (Supertech Instruments UK Ltd., UK, model: TMP-5b and AHP-1)
3. Micro 4 Pump controller for NanoFil syringe (World Precision Instruments, UK, model: UMP3-4)
4. Anesthesia unit (Univentor, model: Univentor 400 Anesthesia Unit with tubes, 10 mL gas-tight glass syringe; anesthesia box, model: 8329001)
5. Air pump (EHEIM GmbH & Co. KG., Germany, model: EHEIM-100)
6. Dual guide LED light illuminator (Zeiss, Germany, model: ZEISS Cold Light Source)
7. Stereo microscope on boom stand (Olympus SZ30, model: SZ3060 SZ-STB1)

## Procedure

- **Viral transduction**
  1. Disinfect the workstation and surgical tools with 70% EtOH. If the procedure is done in the laboratory hood, a 15–30 min UV light can also be used to disinfect the workstation and instruments before the procedure. The syringe can be sterilized by autoclaving or another method recommended by the manufacturer. Wear gloves and wash them with 70% EtOH.
  2. Use scissors to make a mask for the pup. Cut the 200 μL tip so it can be connected to the anesthesia tube. Cut the other side so the pup's nose can fit in the tube. Place the tip connected to the anesthesia tube on the heating pad and fix it on the surface using tape or blu tack.
*Note: We used a 200 μL tip and 1/8 i.d. tubes, but other materials can be used to make the mask, e.g., a 1 mL tip or a plastic Pasteur pipette tip. With these materials, it is also possible to use 1/4 i.d. tubes and fittings to place the mask.*
  3. Prepare the viral vector for injection.
    - Calculate the amount of the viral vector needed for injection, retrieve the proper amount of the aliquots from the -80 °C freezer, and thaw on ice. Always consider the extra amount needed due to possible loss of liquid when filling the syringe.
*Note: Any excess amount of the viral solution should be discarded after the procedure since thaw/freezing cycles are not recommended. Keep the virus-containing liquid on ice.*
    - Using a filtered tip, add glycerol to the viral solution (0.4–4 μL of viral aliquot). If you want to see the liquid with the virus, use glycerol or PBS with dye (see Recipes). Always use a new tip for each aliquot and discard the used tip (into an autoclavable bag or Virkon detergent solution, depending on your safety regulations).
*Note: Step A3b can be skipped. Some researchers find that adding glycerol can protect the AAV and be used for in vivo injections [12,13]. Nevertheless, the absence of glycerol should not be considered dramatic, as many effective protocols do not include this step. The dye is needed for two reasons: first, the liquid containing viral particles is visible during the injection procedure, and second, the injected liquid can be visible within the brain of the neonatal pups through the scalp [14].*
    - Mount the needle to the Nanofil syringe and attach it to the stereotaxic frame.
    - Place the blu tack piece on the heating pad or another surface and place the tube with the virus on it. The tube should stay vertical so the syringe can be filled when attached to the frame.
    - Using the pump, fill the NanoFil syringe with the virus-containing liquid in the amount needed to be injected for all the coordinates for one animal and 100 nL extra to compensate for liquid loss during injection or syringe itself. Use 10 nL/s pump speed for liquid withdrawal.
    - Carefully move the syringe attached to the frame to the side, so the frame is available to place the animal.
  4. Anesthesia of the pup.
    - Turn on a heating pad to keep at 34–35 °C. It should be warm before the animal goes to the anesthesia unit.
    - Adjust the anesthesia unit to provide 4.7%–4.8% isoflurane anesthesia with appropriate air pressure to the unit box. Place a napkin in the box.
    - Place the pup in the anesthesia unit box and keep it closed for at least 2 min until breathing slows down. Switch the unit to provide isoflurane to the tube with a mask.
    - Use a small piece of wipe to make a flat pillow to support the animal's head in a horizontal position. Remove the pup and place it on the heating pad. Adjust its nose to the mask to breathe 4.7%–4.8% isoflurane. Tape the body to the heating pad with a piece of tape. When the animal is fixed on the frame, reduce isoflurane to 2%–2.5%. Check if the animal is anesthetized.
*Caution: Always follow the animal's breathing; it should not be too slow or stop. Do not push too much with the tape on the thorax and do not squeeze the pup. The tape should only be kept so that the head does not move. If the breathing is fragmented, remove the animal from the mask immediately.*
  5. Viral injection.
    - Wash the head with the cotton bud with 70% EtOH.
    - Adjust the light so you can see lambda coordinate as an intersection of thick red vessels (see Figure 1).
      **Figure 1.**
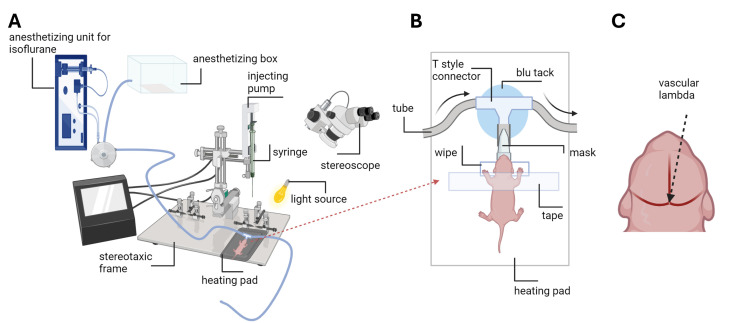
Schematic image of the setup for the viral injection procedure. A. Setup consists of an anesthetizing unit for isoflurane connected to the switcher, directing the flow to the mask or box. The air pump supplying the unit is not shown. The syringe with a needle is assembled to the injecting pump headstage. The headstage is attached to the stereotaxic frame. A heating pad is placed on the stereotaxic frame surface. The pup is positioned on the heating pad, with its nose in the anesthesia mask. A stereoscope is used to see head blood vessels and lambda. The light source is adjusted to light up the pup’s head. B. The pup is placed on the heating pad. The pup’s nose is in the cut pipette tip (mask) connected to the tube system, which provides isoflurane. The tube system is fixed on the surface of the heating pad with the blu tack. A tape is used to gently fix the head of the pup. The head is placed on the flat pillow made from the wipe. The direction of isoflurane flow is demonstrated by arrows. C. The 0 coordinate from vascular lambda is defined as the very middle of the crossing between three branches of blood vessels.
    - Place the syringe tip to ML and AP lambda coordinates to find the 0 coordinate; then, find the coordinate needed for your purpose (for dorsal striatum: AP 2.4; ML ±1.2).
    - Carefully and slowly move the tip down to cross the skin and skull to achieve the correct DV coordinate (for dorsal striatum, DV 2.3 and 2.7). If the tip does not go smoothly through the skull and skin, use a common 30 G syringe needle to make a small hole in the skull before pushing the injecting needle. Start with the lower coordinate.
*Note: There should not be bleeding. In case some minor bleeding occurs, use hydroxy peroxide and cotton bud to remove it. Never contact blood with the needle tip, as it will be clogged.*
    - Inject virus liquid in the needed volume (we used 150 nL). Use 5 nL/s speed. (For dorsal striatum: next, move to the upper coordinate DV 2.3 and inject the same volume again with the same speed.) Wait 5 min before moving the needle up to remove it. Remove it slowly and carefully from the head.
    - Repeat steps A5c–A5e for the next coordinate if needed.
    - Wash the pup’s head with the cotton bud with hydroxy peroxide and then dry it.
  6. Recovery.
    - Remove the animal from the mask but keep it on the heating pad until it reacts to the touch and moves.
    - Place the pup into the dam’s cage.
- **Subcutaneous CNO injections**
  1. Retrieve the vial with CNO from the freezer and thaw it at room temperature.
  2. Separate pups from the dam and transfer them into the empty clean cage.
*Note: When handling active pups, it is recommended to use a plastic tunnel or a cardboard tube rather than cupped hands. See* *https://3rc.org/refined-mouse-handling-overview/operations-and-tunnels* .
  3. Gently transfer a single pup from the cage and place it onto the weighing scale.
**Caution:** Make sure to prevent the pup's escape during the weighing procedure, e.g., by placing a lid on top of the scale.
  4. Place the new needle onto the 25 μL Hamilton syringe and measure the proper volume of CNO according to the weight of the pup, with a final volume of 1 μL per gram of body weight. Control animals receive the injection of sterile saline, 1 μL per gram of body weight.
  5. Administer the injection subcutaneously into the loose skin on the neck of the pup.
**Caution**: Always use different places on the skin for repeating injections.
  6. Following the injection, maintain the needle within the skin for a few seconds before gently withdrawing it to prevent any potential drug leakage from the injection site.
  7. Return the pup to the cage with the dam.
  8. Repeat the procedure for the remaining pups. Change the needle for each injection.

## Data analysis

The effect of the suppression of neuronal activity during the critical developmental period was further investigated by electrophysiological recordings and immunostaining. A detailed description of performed experiments and subsequent analysis can be found in the original research article [15] under the methods section.

## Validation of protocol

This protocol has been used and validated in the research article:

Kokinovic et al. [15]. Spontaneous activity of striatal projection neurons supports maturation of striatal inputs to substantia nigra dopaminergic neurons. eLife.

- First, we validated the expression of fluorescent protein co-expressed with DREADD. A coronal slice of dorsal striatum with the expression of mCitrine is demonstrated in Figure S2B [15].

- It is important to demonstrate the effectiveness of DREADD, which is expected to modify the firing rate of infected neurons. For example, see validation in Figure S2 C of the same publication. In this validation step, we show in vitro the effect of the 10 μM CNO on the firing rate of the striosomal SPN of a 10-day-old mouse injected by DREADD coding AAV at P0. An example trace of membrane potential recording done with whole-cell patch-clamp technique is demonstrated.
- The outcome of the procedure for brain development was analyzed by electrophysiology and microscopy in adult mice, with results shown in Figures 6 and 7 of [15].

## General notes and troubleshooting

**General notes**

1. Always check and follow the safety rules of your research area when handling viruses and GMO mice.
2. The procedures in this protocol require appropriate training and animal license.
3. Avoid contamination when more than one virus is used in the same setup by using different syringes for each or replaceable glass pipettes for each virus to avoid contact between the virus and the syringe. Always change filtered tips when adding glycerol or dye to each virus.
4. If more than one virus needs to be injected, they can be mixed and injected at once. The increase in the volume of injected liquid should be considered.

**Troubleshooting**

Problem 1: The NanoFil syringe is not injecting.

Possible cause: The tip of your needle is clogged by blood cells or another dried organic liquid.

Solution: Try to unclog the needle with a thin wire, then wash and sterilize it or simply replace it with a new one.

Problem 2: Unspecific signal in the brain tissue during validation.

Possible cause: Contamination of syringe, needle, or dye stock by another virus.

Solution: Clean and sterilize the syringe and needle and make another dye stock. Use only your own syringe, needle, and stocks.

Problem 3: No expression of the virally delivered construct after injection.

Possible cause(s): The needle did not reach the desired coordinates during injection, and all the liquid went off under the skin or into the brain ventricles; the virus prep is spoiled (i.e., it was kept at room temperature for a long time, exposed to UV light, or subjected to freezing/tawing cycles); reasons associated with construct and experiment design, such as promoter/enhancer, Cre-dependence, etc.

Solution(s): Use dye to visualize the virus-containing liquid during the injection procedure and verify that it goes inside the brain. Try another aliquot/prep for the virus injection. Check your experiment design and animal genotype.
